# Daily Use of Energy Management Strategies and Occupational Well-being: The Moderating Role of Job Demands

**DOI:** 10.3389/fpsyg.2017.01477

**Published:** 2017-08-31

**Authors:** Stacey L. Parker, Hannes Zacher, Jessica de Bloom, Thomas M. Verton, Corine R. Lentink

**Affiliations:** ^1^School of Psychology, University of Queensland, Brisbane QLD, Australia; ^2^Institute of Psychology, Leipzig University Leipzig, Germany; ^3^School of Management, Queensland University of Technology, Brisbane QLD, Australia; ^4^Institute for Advanced Social Research, University of Tampere Tampere, Finland; ^5^Department of Psychology, University of Groningen Groningen, Netherlands

**Keywords:** energy management, emotional exhaustion, job satisfaction, momentary recovery, job demands, prosocial behavior, organizing behavior, meaning making

## Abstract

We examine the relationships among employees’ use of energy management strategies and two occupational well-being outcomes: job satisfaction and emotional exhaustion. Based on conservation of resources theory, it was hypothesized that employees with high job demands would benefit more from using energy management strategies (i.e., including prosocial, organizing, and meaning-related strategies), compared to employees with low job demands. We tested this proposition using a quantitative diary study. Fifty-four employees provided data twice daily across one work week (on average, 7 daily entries). Supporting the hypotheses, prosocial energy management was positively related to job satisfaction. Moreover, employees with high job demands were less emotionally exhausted when using prosocial strategies. Contrary to predictions, when using organizing strategies, employees with low job demands had higher job satisfaction and lower emotional exhaustion. Under high job demands, greater use of organizing strategies was associated with lower job satisfaction and higher emotional exhaustion. Finally, use of meaning-related strategies was associated with higher emotional exhaustion when job demands were low. With this research, we position energy management as part of a resource investment process aimed at maintaining and improving occupational well-being. Our findings show that this resource investment will be more or less effective depending on the type of strategy used and the existing drain on resources (i.e., job demands). This is the first study to examine momentary effects of distinct types of work-related energy management strategies on occupational well-being.

## Introduction

At work, employees engage in a variety of strategies to help restore depleted energy, maintain energy expenditure, or activate energy reserves, in order to continue with work tasks and to protect or enhance their well-being. Energy management strategies refer to concrete activities which employees deliberately engage in to keep their energy levels high throughout the working day (Fritz et al., 2011). Researchers investigating human energy at work have examined a range of indicators of “energy,” which typically include indicators of occupational well-being, such as higher positive affect, vitality, and affective work engagement, as well as lower fatigue, emotional exhaustion, and health complaints (Quinn et al., 2012). Although there is growing support for the usefulness of some forms of energy management (e.g., micro breaks for well-being), other types of strategies have received scant empirical attention.

Thus, the first goal of the current study is to investigate how daily use of different types of *work-related* energy management strategies are associated with daily indicators of occupational well-being (i.e., job satisfaction and emotional exhaustion). Job satisfaction has been defined as a positive affective state that is linked to employees’ appraisals of their job experiences as enjoyable and pleasant (Locke, 1976). In contrast, emotional exhaustion is a negative affective state associated with feelings of depletion and fatigue due to the pressures of work (Maslach et al., 2001). Daily assessments of job satisfaction and emotional exhaustion can be categorized as “emotional energy” or “energetic activation” (or the lack thereof, in the case of exhaustion; (Quinn et al., 2012).

**FIGURE 1:**
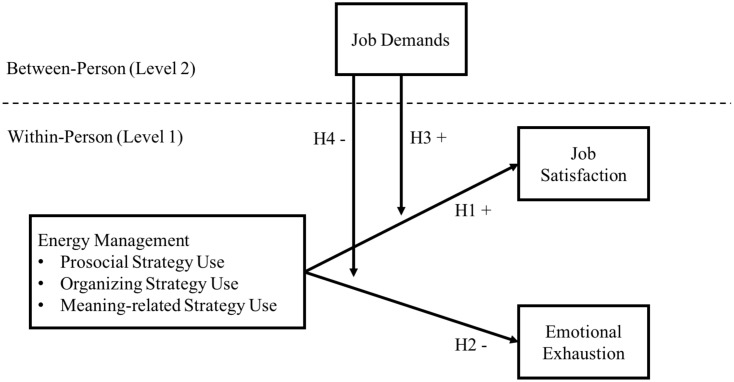
Hypothesized relationships between use of energy management strategies and occupational well-being outcomes moderated by job demands.

The second goal of this study is to investigate employees’ chronic level of job demands as a boundary condition to the effectiveness of energy management. Prior research on energy management has largely failed to examine boundary conditions of the relationships between the daily use of energy management strategies and occupational well-being outcomes. The effectiveness of energy management strategies should be dependent on the level of stress employees are already operating within. Drawing on conservation of resources (COR) theory (Hobfoll, 2011), we argue that it is under high job demands that energy management is essential for the maintenance and/or improvement of day-to-day occupational well-being. Our conceptual model is depicted in **Figure 1**.

### Energy Management and Occupational Well-being

According to Trougakos and Hideg (2009), momentary recovery can occur in two ways: either by stopping a task that depletes regulatory resources or by engaging in a preferred behavior that is enjoyable and therefore energizing. Other classifications of energy management describe two distinct classes of strategies: taking micro breaks and using work-related strategies (see Fritz et al., 2011; Zacher et al., 2014). Based on writings by Loehr and Schwartz (2003), Fritz et al. (2011) suggested that energy management can be categorized based on four different themes; including physical (e.g., sustenance), relational (e.g., prosocial helping), mental (e.g., planning and organizing), or spiritual (e.g., reflecting on meaning). Each theme represents a form of “energy.” However, Quinn et al. argue against these themes (or forms) of energy, specifying that human energy is physical and/or emotional. According to this conceptualization, *energetic activation* (also called emotional energy) is the “energy” construct, and only the *resources we put to use* to protect or enhance energy, that being the strategies we implement, take conceptually distinct forms (see Quinn et al., 2012). Thus, we consider that energy management strategies target one’s subjective sense of well-being at work, that being their energetic activation (e.g., enhanced job satisfaction and reduced emotional exhaustion) and that the strategies themselves can be broadly classified into two classes of strategies: micro-breaks vs. work-related strategies.

The first class of strategies, taking micro breaks (e.g., having a snack, going for a brief walk, surfing the web, talking to a colleague), enables momentary detachment from work and then energy recovery through this brief respite. This perspective assumes, based on ego depletion theory (Muraven and Baumeister, 2000) and COR theory (Hobfoll, 1989), that energy is a limited resource and that work depletes this resource (Meijman and Mulder, 1998; Trougakos and Hideg, 2009; Quinn et al., 2012). The second class of strategies are work-related strategies (e.g., switching work tasks, reflecting on the meaning of work, helping a colleague with their work), which involve switching up one’s tactics associated with how to do work or what to make of work. These strategies do not necessarily assume that energy is a limited resource. Rather, based on broaden and build theory (Fredrickson, 2001) and self-determination theory (Ryan and Deci, 2000), here, energy can be generated through a variety of tactics which might include engagement in pleasant activities that enhance intrinsic motivation, but also through engagement with work in a way that satisfies basic psychological needs (see also Quinn et al., 2012, for a review).

There is growing empirical evidence supporting the importance of breaks during the work day for improving one’s energy. For example, researchers usually find “energy gains” on measures of occupational well-being when employees take a variety of micro breaks throughout the day (Zacher et al., 2014), take relaxing lunch breaks (Trougakos et al., 2008, 2014; Krajewski et al., 2010; Sianoja et al., 2017), take breaks early in the day that involve preferred activities (Hunter and Wu, 2016), take smartphone breaks (i.e., browse the internet or use social media) or have a chat with a colleague (Rhee and Kim, 2016).

In contrast, there is conflicting evidence on the utility of work-related strategies as a form of energy management at work. This could be due to limited investigation into the efficacy of different types of work-related strategies for restoring, maintaining, or enhancing energy. It also could be due to the research methodologies used in the few studies conducted to date. For instance, Zacher et al. (2014), in an experience sampling study, found no benefit of momentary use of work-related strategies for improving hourly reports on vitality and fatigue. However, using cross sectional survey designs, researchers do find benefits to use of work-related strategies. For example, higher vitality (Zacher et al., 2014), and also better ratings of health, work engagement, and performance (de Bloom et al., 2015), especially when work-related strategies are used in combination with micro breaks (Kinnunen et al., 2015).

Fritz et al. (2011) reported the first investigation into these two classes of energy management strategies (i.e., micro breaks and work-related strategies) using a cross-sectional survey of knowledge workers, and found that a few specific work-related strategies (e.g., related to being prosocial, finding meaning, and learning) were associated positively with vitality. We believe further systematic research using experience sampling designs is needed to provide a more complete picture of how work-related strategies might be most effectively utilized for energy restoration or gain. It is important to measure the use of strategies *in the moment* (i.e., as it happens), so that we can capture the natural base rates (i.e., frequency of use) and temporal variation in energy management day-to-day. This way, the immediate effects of strategy use on indicators of occupational well-being can be assessed.

So far, researchers have examined the effects of work-related strategies using an overall composite of all known/common strategies (see Zacher et al., 2014; de Bloom et al., 2015; Kinnunen et al., 2015) or as 20 single items (see Fritz et al., 2011). These approaches are problematic, as they may lead to inconsistent findings within the literature related to the efficacy of work-related strategies. In the only experience sampling study conducted to date, an overall composite of work-related strategies produced no “energy gains” (or losses) on indicators of occupational well-being (Zacher et al., 2014). However, if we examine the momentary effects of distinct types of work-related energy management strategies, it is possible that such beneficial effects could be revealed. This detailed analysis of work-related strategies would allow for observation of the unique positive (or even negative) effects of certain strategies (without averaging out unique effects in the analysis). Here, by measuring distinct strategies, we aim to better “unpack” work-related strategies as a form of daily energy management at work.

In determining which types of work-related energy management strategies to investigate, we focused on common and proactive strategies identified through the exploratory work of Fritz et al. (2011) and Zacher et al. (2014). Prosocial strategies entail prosocial interactions with other people at work, such as helping a co-worker. Organizing strategies refer to future-oriented behaviors, such as making to-do lists and setting new goals. Finally, meaning-related strategies include behaviors that help employees see the broader meaning of their work, such as reflecting on one’s work tasks (Fritz et al., 2011). Thus, in this study we focus on managing energy by engaging with work in a different way; by helping others (i.e., prosocial strategies), planning and organizing it (i.e., organizing strategies), or reflecting on its meaning and importance (i.e., meaning-related strategies). In selecting these strategies, we also have responded to calls for more research on energy management strategies related to interpersonal processes at work, planning, and reflection (Schippers and Hogenes, 2011). In the next section, we outline how and when these work-related energy management strategies will enhance occupational well-being, guided by COR theory.

### A Conservation of Resources Approach to Energy Management

According to COR theory, individuals seek to create a world that provides them with pleasure and success, and they do this by acting to enhance the likelihood of maintaining and increasing their resources (Hobfoll and Leiberman, 1987; Hobfoll, 1989, 2001; Westman et al., 2005; Hobfoll, 2011). Resources are broadly defined as objects, states, conditions, and other things individuals value (Hobfoll, 1989; Halbesleben et al., 2014). More recent review and integration of the COR literature has positioned time away from work and recovery experiences as energy resources (Halbesleben et al., 2014). We argue that within-day energy management strategies would also fall under this energy resource category. However, such strategies are only an energy resource when “put to use” effectively (Quinn et al., 2012). In this way, energy management is part of a process of resource investment aimed at improving one’s occupational well-being. To gain resources (i.e., occupational well-being) one needs to invest resources (i.e., by implementing energy management strategies).

As such, using energy management strategies constitutes a resource investment process that should have positive short-term effects on occupational well-being, because these strategies may stop the depletion of resources and also have the potential for energizing effects (Trougakos and Hideg, 2009; Schmitt et al., 2012). Each of the types of work-related strategies we investigate (i.e., prosocial, organizing, and meaning-related) has the potential to restore and/or enhance energy, as each involves a change to work tasks (Trougakos and Hideg, 2009), either objectively or subjectively. For instance, use of organizing strategies could improve occupational well-being by helping one to better manage their time and direct their effort, thereby enhancing motivation and stopping the depletion of resources (Trougakos and Hideg, 2009). Use of meaning-related strategies might remind one of how important the work is (Schippers and Hogenes, 2011), thereby providing the motivation to re-engage with it in a positive way that is energizing and not depleting. Finally, use of prosocial strategies can also be motivating, thereby improving the well-being not just of the recipient but also the helper (Weinstein and Ryan, 2010). Therefore, we expect that use of each of the three work-related strategies is positively related to employees’ daily job satisfaction and negatively related to their daily emotional exhaustion.

*Hypothesis 1:* The daily use of (a) prosocial, (b) organizing, and (c) meaning-related strategies is positively related to daily job satisfaction.
*Hypothesis 2:* The daily use of (a) prosocial, (b) organizing, and (c) meaning-related strategies is negatively related to daily emotional exhaustion.

Switching up how to do work or what to make of work has the potential to activate one’s energy, but this depends on effectively putting resources to use (Quinn et al., 2012). As such, we also draw on COR theory to explain how “energy gains” to occupational well-being (i.e., enhanced satisfaction and reduced exhaustion) during the work day will depend on the level of job demands employees already operate under. In life, we are often faced with situations that have the potential to lead us to gain or lose resources. Quantitative job demands describes the extent to which employees have to complete a lot of work tasks within a short period of time (Spector and Jex, 1998). Working under chronically high job demands is one of those situations, which has been found to erode job satisfaction and cause emotional exhaustion (Örtqvist and Wincent, 2006).

Individuals allocate or invest their resources (e.g., through energy management) in order to protect current resources and acquire new resources (e.g., occupational well-being; Quinn et al., 2012). The boundary conditions to this process are best described through three main principles of COR theory (Westman et al., 2005; Hobfoll, 2011; Halbesleben et al., 2014). The first principle is *the primacy of resource loss*. According to this principle, resource loss is highly salient and has deleterious consequences for one’s well-being. The second principle is that of *the motivation of resource investment*. To gain resources, protect resources, or to recover from resource loss, individuals recognize they must invest resources. However, one related corollary of this principle is that those with greater resources are less vulnerable to resource loss and more capable of resource gains. Finally, the third principle is the *paradoxical integration of principles one and two*, in that although resource loss is more salient than resource gain, the motivation to invest resources in order to gain resources increases under situations of resource loss (Hobfoll, 2011). As such, under conditions of great stress or challenge, individuals are motivated to employ other resources to offset or minimize the resource losses they might incur, and it is in these situations in which they might possibly reap the most benefits. This aspect of the theory also relates to the *substitution hypothesis* (also referred to as *resource compensation*), whereby when a resource is lacking or threatened an individual might be able to compensate by leveraging their access to another resource (Hobfoll and Leiberman, 1987). In this way, we argue that use of energy management strategies under high job demands is aimed at leveraging one’s momentary energy resources to mitigate further losses to occupational well-being (and/or to generate gains to occupational well-being). In this way, we believe that chronic job demand is experienced as an intense and salient threat of resource loss, and as such it is under these conditions that use of energy management strategies are needed the most and have the potential to provide the most benefit to occupational well-being.

Many theories of human energy (and motivation) suggest that energetic activation will only occur when the situation is “just right” or conducive enough (Quinn et al., 2012). As such, we expect that when employees who are operating under high chronic job demands put their energy management strategies to use, it is in this situation that gains to occupational well-being should be observed, as this is the employee group that has the most at stake and really needs to manage their energy effectively. Thus, we expect that the use of energy management strategies is more beneficial for occupational well-being when job demands are high.

*Hypothesis 3:* The positive relationships of the daily use of (a) prosocial, (b) organizing, and (c) meaning-related strategies with daily job satisfaction are stronger among employees with high compared to low job demands.
*Hypothesis 4:* The negative relationships of the daily use of (a) prosocial, (b) organizing, and (c) meaning-related strategies with daily emotional exhaustion are stronger among employees with high compared to low job demands.

## Materials and Methods

### Participants and Procedure

To test our hypotheses, we conducted a 1-week quantitative diary study where employees completed twice daily assessments of their momentary energy management strategies and occupational well-being. Participants in this quantitative daily diary study were 54 administrative and academic employees of a university in the Netherlands. This sample size is adequate for detecting small to medium effect sizes in multilevel analyses, including testing for cross-level moderation (Scherbaum and Ferreter, 2009). Of the participants, 43 were female and 11 were male. Participants ages ranged from 23 to 63 years, with a mean age of 36.53 years (*SD* = 13.37). The majority of participants held a university degree (61.1%). Job tenure ranged from a few months to 22 years, with a mean of 4.25 years (*SD* = 4.92).

Participants were recruited in person by walking into offices and inviting employees to participate in a daily diary study on their work experiences. Employees (*N* = 105) who initially agreed to take part in the study received an email the next day with a link to a baseline survey that included measures of job demands and demographic characteristics. In the following week beginning on a Monday, employees received emails every day at 11 a.m. and at 3 p.m. with a link to a digital survey on energy management strategies and occupational well-being, to be completed before lunch and before the end of the work day, respectively. Only responses received within 2 h after the emails were sent were included in the analyses. The data collection period ended on Friday. We included only employees who completed the baseline survey and at least three daily surveys. The final sample of 54 employees completed 386 daily surveys (on average, 7.15 completed daily surveys, with a minimum of three and a maximum of 10 daily surveys), which is a typical level of compliance with the research procedure for this type of research design (Ohly et al., 2010).

Data for this study was collected as part of a larger research project and one article with a completely different research question than the current article has already been published (Zacher, 2015). None of the variables that are included in the current study were also included in the published article. This study was reviewed and approved by the Ethical Committee Psychology at the University of Groningen (Netherlands^1^). All participants gave written informed consent in accordance with the Declaration of Helsinki.

### Measures

All items and factor loadings from a multilevel confirmatory factor analysis (see Statistical Analyses) are shown in **Table 1**.

**Table 1:** Items and factor loadings.

Item	Variable	Factor loading
Making time to show gratitude to someone I work with.	Prosocial strategies	0.67
Doing something that will make a colleague happy.	Prosocial strategies	0.87
Offering help to someone at work.	Prosocial strategies	0.73
Checking and updating schedule.	Organizing strategies	0.88
Making a to-do list.	Organizing strategies	0.92
Setting a new goal.	Organizing strategies	0.77
Reflecting on the meaning of my work.	Meaning-related strategies	0.93
Reflecting on how I make a difference at work.	Meaning-related strategies	0.96
Focusing on what gives me joy at work.	Meaning-related strategies	0.75
I feel fairly well satisfied with my job.	Job satisfaction	0.86
I am enthusiastic about my work.	Job satisfaction	0.90
This work day seems like it will never end. (reversed)	Job satisfaction	0.55
I find real enjoyment in my work.	Job satisfaction	0.92
I consider my job rather unpleasant. (reversed)	Job satisfaction	0.54
I feel emotionally drained from my work.	Emotional exhaustion	0.92
I feel used up due to my work.	Emotional exhaustion	0.92
I feel burned out from my work.	Emotional exhaustion	0.87
How often does your job require you to work very fast?	Job demands	0.80
How often does your job require you to work very hard?	Job demands	0.97
How often is there a great deal to be done?	Job demands	0.70


*Energy management items were adapted from Fritz et al. (2011), job satisfaction items were adapted from Judge et al. (1998), emotional exhaustion items were adapted from Maslach and Jackson (1981), and job demands items were adapted from Spector and Jex (1998).*

#### Energy Management Strategies

Use of prosocial, organizing, and meaning-related energy management strategies was measured in the daily surveys with items from Fritz et al. (2011) and Zacher et al. (2014) that were adapted to the daily level (“This morning/afternoon at work, I managed my energy by…”). Participants indicated how frequently they had made use of each strategy on a 5-point scale, ranging from 1 (*never*) to 5 (*very often*). Cronbach’s alphas were 0.84, 0.89, and 0.91 for prosocial, organizing, and meaning-related strategy use, respectively.

#### Job Satisfaction

Job satisfaction was measured in the daily surveys with five items adapted from a widely used, well-validated scale by Judge et al. (1998; see **Table 1**). Items were answered on 5-point Likert scales, ranging from 1 (*strongly disagree*) to 5 (*strongly agree*). Cronbach’s alpha was 0.86.

#### Emotional Exhaustion

Emotional exhaustion was measured with three items adapted from a widely used and well-validated scale from Maslach and Jackson (1981; see **Table 1**). Items were answered on 5-point Likert scales, ranging from 1 (*strongly disagree*) to 5 (*strongly agree*). Cronbach’s alpha was 0.93.

Previous research at the within-person level has shown that these two indicators of occupational well-being (i.e., job satisfaction and emotional exhaustion) fluctuate substantially within-persons over time (Ilies and Judge, 2004; Grech et al., 2009; Schmitt et al., 2012), and are therefore suited to investigation with an experience sampling research design.

#### Job Demands

Quantitative job demands were measured in the baseline survey with three items from a scale developed and validated by Spector and Jex (1998; see **Table 1**). The response scale ranged from 1 (*never*) to 7 (*daily*). Cronbach’s alpha was 0.88.

### Statistical Analyses

As the data collected for this study had a multilevel structure, we used hierarchical linear modeling (HLM) software to analyze it (Raudenbush and Bryk, 2002). The between-person variable (job demands) was centered at the grand (or sample) mean, and the within-person predictors (energy management strategies) were centered at each participant’s mean (Ohly et al., 2010; Aguinis et al., 2013). We probed significant interaction effects using the methods set out by Preacher et al. (2006).

We examined the factor structure of all items by computing multilevel confirmatory factor analyses. A model with five factors on the within-person level (three energy management strategies, job satisfaction, and emotional exhaustion) and one factor at the between-person level (job demands) had a very good fit with the data [χ^2^(109) = 187.542, *p* < 0.001; CFI = 0.971; TLI = 0.963; RMSEA = 0.043; SRMS_within_ = 0.057]. In contrast, a one-factor model did not fit the data well [χ^2^(90) = 2208.582, *p* < 0.001; CFI = 0.160; TLI = -0.008; RMSEA = 0.247; SRMR_within_ = 0.263].

## Results

Descriptive statistics and correlations are shown in **Table 2**. Results of null models showed that 46% of the variance in job satisfaction and 50% of the variance in emotional exhaustion resided at the within-person level. Similarly, the proportions of within-person variance for the energy management strategies ranged from 43% (organizing strategy use) to 59% (prosocial strategy use; see **Table 2**).

**Table 2:** Descriptive statistics and correlations of variables.

Variable	*M*	*SD*	1-ICC	1	2	3	4	5	6
**Between-person variable**									
(1) Job demands	4.73	1.31	–	(0.88)					
**Within-person variables**									
(2) Prosocial strategy use	1.92	0.85	0.59	-0.11	(0.84)	0.03	0.27^∗∗^	0.15^∗∗^	-0.09
(3) Organizing strategy use	2.03	1.01	0.43	-0.03	0.43^∗∗^	(0.89)	0.15^∗∗^	0.01	0.04
(4) Meaning-related strategy use	1.71	0.91	0.44	0.16	0.36^∗∗^	0.37^∗∗^	(0.91)	0.11^∗^	-0.02
(5) Job satisfaction	3.99	0.64	0.46	0.04	0.17	0.06	0.24	(0.86)	-0.57^∗∗^
(6) Emotional exhaustion	1.80	0.80	0.50	0.06	-0.05	-0.15	-0.08	-0.61^∗∗^	(0.93)


*Correlations above the diagonal represent within-person (level 1) relationships of person-mean centered variables (*N* = 386), and correlations below the diagonal represent between-person (level 2) relationships of between-person variables and aggregated daily variables (*N* = 54). 1-ICC = proportion of within-person variance. Reliability estimates (α) are shown in parentheses along the diagonal. *^∗^p* < 0.05, ^∗∗^*p* < 0.01.*

According to Hypothesis 1, the daily use of (a) prosocial, (b) organizing, and (c) meaning-related strategies is positively related to daily job satisfaction. Hypothesis 2 states that the daily use of (a) prosocial, (b) organizing, and (c) meaning-related strategies is negatively related to daily emotional exhaustion. **Table 3** shows the results of two multilevel analyses predicting the occupational well-being outcomes: job satisfaction and emotional exhaustion. Only prosocial strategy use had a positive and significant main effect on job satisfaction (γ = 0.09, *p* = 0.012). The more frequently participants used prosocial energy management strategies, the higher their levels of job satisfaction. None of the other strategies significantly predicted job satisfaction. In addition, and contrary to expectations, there were no significant main effects of work-related energy management strategies on emotional exhaustion. Thus, Hypothesis 1 was only partially supported, and Hypothesis 2 was not supported.

**Table 3:** Results of hierarchical linear modeling analyses predicting occupational well-being.

	Job satisfaction γ (*SE*)	Emotional exhaustion γ (*SE*)
Intercept	3.98 (0.07)^∗∗^	1.81 (0.08)^∗∗^
**Between-person predictor**		
Job demands	0.01 (0.05)	0.03 (0.06)
**Within-person predictors**		
Prosocial strategy use	0.09 (0.04)^∗^	-0.09 (0.05)
Organizing strategy use	0.00 (0.04)	0.02 (0.05)
Meaning-related strategy use	0.04 (0.04)	0.04 (0.06)
**Cross-level interactions**		
Prosocial strategy use × Job demands	0.04 (0.03)	-0.07 (0.04)^∗^
Organizing strategy use × Job demands	-0.11 (0.03)^∗∗^	0.13 (0.03)^∗∗^
Meaning-related strategy use × Job demands	0.04 (0.03)	-0.10 (0.04)^∗^


**N* = 386 daily survey responses nested within 54 participants. Unstandardized multilevel modeling coefficients (γ) with standard errors (*SE*) are shown. ^∗^*p* < 0.05, ^∗∗^*p* < 0.01.*

**FIGURE 2:**
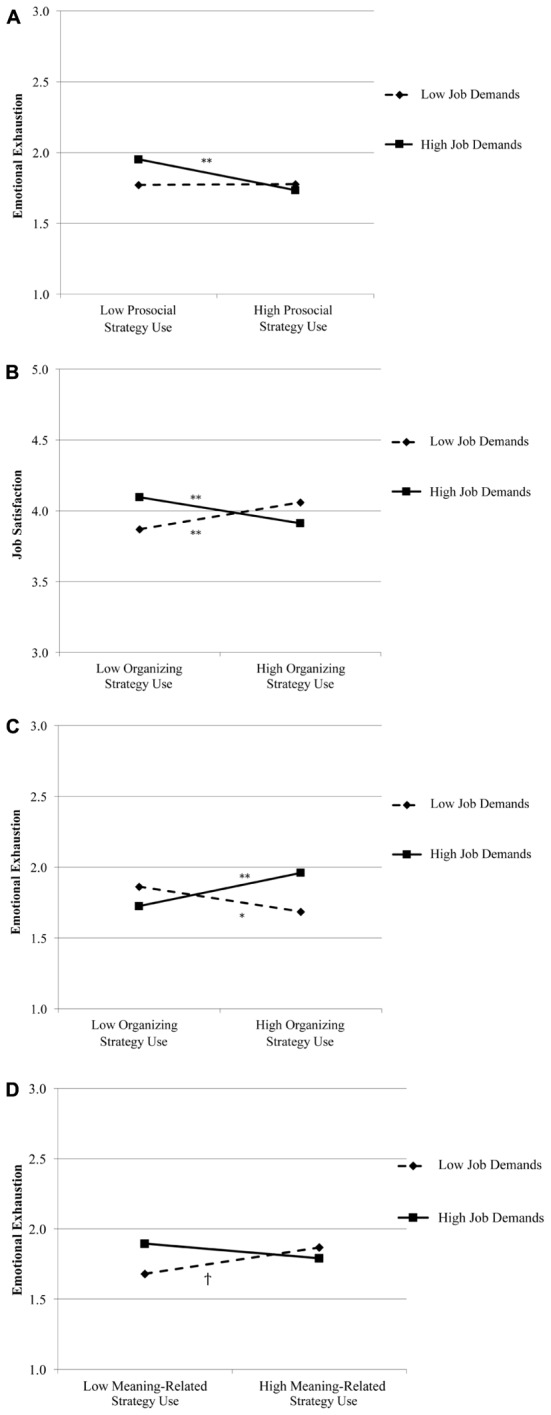
Relationships between use of energy management strategies and occupational well-being outcomes moderated by job demands (^†^*p* < 0.075, ^∗^*p* < 0.05, ^∗∗^*p* < 0.01).

According to Hypothesis 3, the positive relationships of the daily use of (a) prosocial, (b) organizing, and (c) meaning-related strategies with daily job satisfaction are stronger among employees with high compared to low job demands. Hypothesis 4 states that the negative relationships of the daily use of (a) prosocial, (b) organizing, and (c) meaning-related strategies with daily emotional exhaustion are stronger among employees with high compared to low job demands. As shown in **Table 3**, job demands moderated the relationship between prosocial strategy use and emotional exhaustion. We regressed emotional exhaustion on prosocial strategy use at low (i.e., one standard deviation below the mean) and high (i.e., one standard deviation above the mean) levels of job demands. These simple slope analyses showed that the relationship was weak and non-significant at low (γ = 0.004, *p* = 0.950) and negative at high job demands (γ = -0.18, *p* = 0.005; **Figure 2A**). In line with our hypotheses and COR theory, this means that prosocial strategy use decreased exhaustion among employees with high job demands.

Job demands further moderated the relationships between organizing strategy use and both job satisfaction and emotional exhaustion (see **Table 3**). The relationship between organizing strategy use and job satisfaction was positive at low (γ = 0.15, *p* = 0.003) and negative at high job demands (γ = -0.15, *p* = 0.002; **Figure 2B**), meaning that organizing strategy use heighten job satisfaction among employees with low job demands and lowered job satisfaction among employees with high job demands. Similarly, the relationship between organizing strategy use and emotional exhaustion was negative at low (γ = -0.16, *p* = 0.025) and positive at high job demands (γ = 0.19, *p* = 0.003; **Figure 2C**). This suggests that organizing strategy use lowered exhaustion among employees with low job demands and increased exhaustion among employees with high job demands.

Finally, job demands moderated the relationship between meaning-related strategy use and emotional exhaustion, such that the relationship was positive at low (γ = 0.17, *p* = 0.064) and weak and non-significant at high job demands (γ = -0.09, *p* = 0.148; **Figure 2D**).

Overall, these findings indicate that Hypothesis 4 was supported for prosocial strategy use only, because only prosocial strategy use was negatively associated with exhaustion when job demands were high. Hypothesis 3 was not supported as the empirical interaction patterns were different from those hypothesized.

## Discussion

The first goal of study was to examine momentary relationships between three types of work-related energy management strategies (i.e., prosocial, organizing, and meaning-related) and occupational well-being (i.e., job satisfaction and emotional exhaustion). The second goal was to investigate employees’ chronic job demands as a boundary condition of these relationships. We expected that use of energy management strategies would be associated with higher job satisfaction and lower emotional exhaustion, and this would be especially so for employees working under high job demands (who should benefit from energy management the most).

We tested these ideas using a 1-week quantitative diary study with employees, so we could tap daily variations in energy management strategy use and the immediate (or momentary) effects of strategy use on our indicators of occupational well-being. Indeed, results showed substantial within-person variation in strategy use and well-being outcomes (between 43 and 61% of the total variance), suggesting there was substantial variation in daily well-being that could potentially be explained by strategy use. However, we found weak support for the main effects of energy management strategy use onto our daily assessments of occupational well-being. Instead, interesting interactive results were revealed regarding the efficacy of different work-related energy management strategies, depending on employees’ level of chronic job demands.

### Benefits of Prosocial Strategies

In line with COR theory, and consistent with previous research on work-related energy management strategies (Fritz et al., 2011; Zacher et al., 2014), prosocial strategy use was positively related to job satisfaction within the work day. In the initial Fritz et al. (2011) study, it was the single items “*do something that will make a colleague happy*” and “*make time to show gratitude to someone I work with*” that were positively related to subjective vitality (but not negatively related to fatigue). Indeed, consistent with these findings, Zacher et al.’s (2014) experience sampling study found that when these two items were combined *post hoc* to form a “prosocial” scale, this was the only indicator of work-related energy management that predicted momentary vitality (i.e., in the hour subsequent to strategy use, after controlling for time of day and vitality in the previous hour). Thus, overall, prosocial strategies, such as helping others and showing compassion at work, appear to improve employees’ “energetic activation” (i.e., vitality and satisfaction) at work, both *in general*, when measured through cross-sectional surveys (Fritz et al., 2011; Zacher et al., 2014), as well as *in the moment*, when collected using experience sampling methodologies (i.e., both hourly, Zacher et al., 2014; and with a daily approach as observed in this study).

In this study, not only was there a positive main effect of prosocial strategy use on daily reports of job satisfaction, in support of our moderation hypotheses there was also an interactive effect, whereby emotional exhaustion was reduced among employees with high job demands who used this strategy. In line with COR theory (Hobfoll, 1989, 2001, 2011) and human energy theory (Quinn et al., 2012), this finding supports the idea that individuals under high job demands (or high threat to their valued resources) benefit when they put their resources to use, or to put it another way, benefit when they make a resource investment in the form of prosocial energy management. According to COR theory, it is under high threat situations that individuals potentially reap the most gains. In this instance, through the use of prosocial energy management they experience less emotional exhaustion, despite their high job demands. These findings are also in line with research more broadly demonstrating that helping others improves one’s own well-being, and that this association is because altruistic acts satisfy basic psychological needs and improve autonomous motivation (Weinstein and Ryan, 2010).

These findings also are informed by recent work on the effects of organizational citizenship behavior. For example, researchers have found that employees who are already chronically emotionally exhausted and also engaging in depleting surface acting are less likely to engage in organizational citizenship behaviors (Trougakos et al., 2015). Others have shown that helping others at work is depleting and increases self-serving behaviors, especially for those who have a prevention regulatory focus (Gabriel et al., 2017). Here, the idea is that too much ‘work pressure’ depletes one’s resources and thereby their ability to engage in prosocial helping. We did not find this to be the case in our study, instead finding that those with chronically high job demands who used prosocial strategies on a daily basis reported lower daily levels of emotional exhaustion. There is an ongoing debate in the organizational citizenship literature as to whether citizenship behaviors are draining or enriching of people’s resources. The prevailing perspective is that engaging in helping has the potential to deplete willpower and other personal and energy resources (see Lanaj et al., 2016). However, in a recent experience sampling study, it was revealed that ‘high performers’ who engaged in organizational citizenship behaviors experienced greater vigor (through enhanced meaningfulness of work; see Fu Lam et al., 2016). Consistent with Fu Lam et al. (2016) our findings with prosocial helping as an energy management strategy support an enrichment perspective, and also suggest that personal and energy resources are abundant. As such, engaging in altruistic acts (i.e., helping others at work) can improve one’s own well-being because these behaviors are in and of themselves (or upon reflection; see Lanaj et al., 2016) energizing or replenishing.

Indeed, there is such growing interest in this enrichment perspective of citizenship behavior, that scholars have begun to examine new energy constructs, these being relational energy (Owens et al., 2016) or collective energy (Cole et al., 2012), which are described as forms of energy garnered from positive interactions with leaders and/or team mates. Guided by Quinn et al. (2012), we caution researchers in regard to the proliferation of different energy constructs. We consider that examination of different relational or collective forms of energy might conflate cause (e.g., positive interactions with a leader) and effect (e.g., energetic activation from those interactions).

Our quantitative daily diary study also revealed emergent findings involving the other energy management strategies investigated.

### When to Use Organizing Strategies

Contrary to hypotheses, there were no main effects of organizing strategies on indicators of occupational well-being. It is hard to determine, given prior research on use of this strategy, whether this lack of main effects is reliable or not. In the Fritz et al. (2011) study, most of the single items related to organizing did not correlate with vitality or fatigue, only the item “*setting a new goal*” was positively associated with vitality, which we included in our measure of organizing. In other research that has used these original items from Fritz et al. (2011) items were grouped together as “work-related strategies,” including both items to do with setting goals with items to do with prosocial helping of colleagues (e.g., de Bloom et al., 2015), or items to do with making lists and setting goals with items to do with reflecting on the meaning of work (e.g., Kinnunen et al., 2015). This makes it difficult to ascertain the unique effects of organizing strategies (as compared to other work-related strategies). In other research, where follow-up analyses teased strategies apart *post hoc*, organizing strategies had no overall effect on vitality or fatigue, only a few single items had beneficial momentary effects, including making lists, which both decreased fatigue and increased vitality, and updating one’s schedule, which increased vitality (Zacher et al., 2014).

Our interactive effects involving organizing as an energy management strategy revealed that using such strategies, like making to-do lists, switching tasks, and setting new goals, was only beneficial among employees with low job demands. In contrast, organizing strategies appeared to reduce occupational well-being among employees with high job demands. These findings are contrary to the main principles of COR theory, however, do speak to a related corollary of these principles, that being that those who lack resources are more vulnerable to further resource loss (Hobfoll, 1989, 2001, 2011). Moreover, these findings are consistent with related research, and suggest that the utility of organizing strategies would be highly dependent on context.

In cognitive psychology, for example, experimental research on task-switching has revealed that frequent task switching can impair task performance through momentary prospective memory failure, because this behavior is effortful and draining on memory resources (Finstad et al., 2006). For employees with high job demands, updating a schedule and making a to-do-list may actually increase their already high-perceived job demand (i.e., is stress arousing), because these actions remind them in the moment that they have too much work to do. This is quite interesting, when one considers that organizing strategies could be akin to problem-focused coping efforts. Interestingly, problem-focused coping has been found to be counter-productive when faced with high demands, because such behavior offers no detachment from the source of stress (Korner et al., 2012).

According to Quinn et al. (2012) energy management strategies that are familiar should not be draining, as one has practice at using them. However, scholars also have reasoned that work-related strategies, like organizing, might involve a continued expenditure of resources through little or no respite from work (Trougakos and Hideg, 2009), which, in turn, leads to lower momentary well-being. Alternatively, that organizing strategies might unfold their beneficial effects in the longer-term because the benefits take a longer amount of time to be realized (e.g., across days or weeks; Zacher et al., 2014). It might not be that the strategy itself that is draining or energizing, but as per our findings, rather it depends on the pre-existing level of job demands. As such, we can very tentatively conclude that employees operating under low job demands, who are experiencing less pressure, may be best positioned to benefit from the challenging yet potentially energizing effects of organizing as an energy management strategy. However, further research is needed to determine exactly when and how such strategies can be used to maintain and improve energy (especially over the longer term).

### Potential Costs of Meaning-Related Strategies

We found no main effects of meaning-related strategy use on our indicators of occupational well-being. In their sample of knowledge workers, Fritz et al. (2011) found the single items of “*focus on what gives me joy at work*,” “*reflect on how I make a difference at work*,” and “*reflect on the meaning of my work*,” each positively related to subjective vitality. We integrated these items into our daily measurement of meaning-related energy management, so were surprised not to see a direct effect. However, these strategies might not have immediate momentary benefits. Indeed, in the Zacher et al. (2014) experience sampling study, only one of these items positively related to hourly reports on vitality (e.g., *focus on what gives me joy at work*); whereas, a composite of the three items called *reflection*, when measured in a general survey, correlated positively with general vitality [as observed in Fritz et al. (2011) who also used a cross-sectional research design], but did not correlate with well-being in the hourly assessments. This lack of immediate effect in diary study research on energy management is consistent with recent research on positive work experiences and positive work reflection (Meier et al., 2016). It might be that meaning-related energy management strategies have a delayed effect or that these strategies are more effective when put into use in the evenings after work (not during work). Moreover, scholars calling for more research on the role of reflection in energy management have suggested that it is reflection on long-term goals (rather than momentary or short-term goals) that might have the most impact on energy (Schippers and Hogenes, 2011). Unpacking the temporal processes involved in meaning-related strategies would be a worthwhile direction for future research, including investigating the use of meaning-related (or reflection) energy management strategies outside of work time.

### Theoretical and Practical Implications

Our research contributes to COR theory and energy management theory. Here, in line with COR theory (Hobfoll, 2011; Halbesleben et al., 2014), we have positioned energy management strategy use as part of a resource investment process aimed at maintaining and improving occupational well-being. This resource investment will be more or less effective depending on the type of strategy used and the existing drain on resources (i.e., job demands). Our findings are also consistent with recent theoretical arguments about the reciprocal and dynamic nature of human energy at work (Quinn et al., 2012), these arguments being that it is the discrepancy between job demands and *use of* resources to meet those demands that drives energetic activation (i.e., greater job satisfaction and less emotional exhaustion).

We found that not all strategies are created equal and the effectiveness of certain work-related energy management strategies depends on the context, that is, the employees’ job demands. We found that prosocial strategies can lessen exhaustion for those with high demands. In contrast, organizing strategies are only beneficial, for both satisfaction and exhaustion, in jobs with low demands (and detrimental in jobs with high demands). Also, reflecting on the meaning of one’s work might have a potentially backfiring effect for those without much job pressure. Trougakos and Hideg (2009) theorized that is the *nature* of momentary recovery that has the potential to improve affective and regulatory resources, and subsequently well-being and work task performance. Accordingly, to be effectively *put to use*, work-related energy management strategies need to both increase intrinsic motivation and decrease work intensity (lessen the momentary drain on regulatory resources and therefore the need for respite; Quinn et al., 2012). Following on from this, one reason why prosocial strategy use seems to be well supported as an adaptive energy management strategy could be because it activates both mechanisms, both affective and regulatory intermediary resources. It is possible that organizing and meaning-related strategies do not positively trigger both types of mechanisms during the resource investment process. Further research is needed to unpack the mechanisms through which work-related energy management strategies improve momentary occupational well-being, and whether these mechanisms can explain why certain contexts have the potential to undermine the efficacy of certain energy management strategies.

Overall, these findings suggest that one way to improve occupational well-being at work is to create more opportunity for employees to act altruistically by engaging in prosocial helping behavior. This opportunity to act could be motivated through work redesign and or culture change (Grant, 2007), as well as through team and leadership development (Cole et al., 2012). One final practical implication of this research could be training and development interventions on the *what* and *when* of energy management at work, for example training for employees under high demands on what strategies are most effective (i.e., use prosocial but limit the regular use of organizing for momentary well-being gains).

### Limitations and Future Research Directions

The two key limitations of this research are the small sample size and that the data is self-report, which enhances the risk of common method bias. However, the use of a quantitative daily diary design over the course of one working week does mitigate this issue (Beal, 2015). Future research is needed to replicate these findings in larger and more heterogeneous samples (e.g., blue-collar workers, workers with varying levels of job autonomy).

Moreover, it is important to examine how long lasting the positive and negative effects of using work-related energy management strategies are for occupational well-being. Cross-lagged analyses across shorter time intervals, such as a few hours (Zacher et al., 2014), are also needed to probe the causal direction of relationships. It may be possible that levels of occupational well-being, in combination with job demands, predict employees’ use of certain energy management strategies and not vice versa.

Another potential criticism might be that we focused on job demands in an initial baseline survey approximately 1 week before the diary study, rather than assessing momentary fluctuations in job demands during the work week. The reason we examined the interactive effect using chronic job demands (i.e., baseline assessment of a between-persons variable) and not momentary job demands (i.e., daily assessments) is because we believed that chronic job demands would be experienced as a more intense and salient threat of resource loss, and as such would be when we would observe the benefit of energy management. Daily fluctuations in demands might not be threatening to resources, but experienced as natural ebbs and flows in task demands. In this way, daily variations in demand might not be detrimental enough for energy management to really matter for occupational well-being. This being said, we did include a daily assessment of task demands in our experience sampling surveys, so that we could compare chronic demands with daily demands as the moderating variable. Importantly, this follow-up analysis with daily demands revealed no significant effects (i.e., no main or interactive effects), thus supporting the important role of chronic job demands in determining when energy management is important for occupational well-being.

In this study, an interactive effect revealed that use of meaning-related strategies appeared to increase emotional exhaustion among employees with low job demands. The use of meaning-related strategies may exhaust employees with low demands because, upon reflection, they may realize the lack of motivating factors (e.g., job challenge) in their daily work. As such, future research might examine different types of job stressors (LePine et al., 2005) or job stressor appraisals (LePine et al., 2005; Searle and Auton, 2014) as boundary conditions to the utility of energy management strategies.

Another interesting future research direction would be examining the effect of use of energy management strategies at work on after work recovery (e.g., Demerouti et al., 2012). Initial cross-sectional investigations into this issue suggest that recovery processes during and after work mutually reinforce each other, potentially resulting in gain spirals (de Bloom et al., 2015). In order to empirically test such gain spirals, cross-lagged experience sampling research designs could be used in future research.

It also may be important to examine additional boundary conditions. For example, experience sampling research has shown that a good night’s sleep in combination with use of micro breaks during the work day can enhance work engagement (Kühnel et al., 2017). Indeed, a cross-sectional study using latent profile analysis found that job autonomy and social support were positively associated with the use of work-related energy management strategies (Kinnunen et al., 2015). Such job resources might better enable adaptive use of work-related energy management strategies in the heat of the moment. It also underscores the importance of healthy job designs, which better enable employees to actively manage their energy at work.

Finally, in relation to the use of prosocial energy management strategies, with our measure we do not know exactly who is the target of employee helping behavior (i.e., a colleague, leader, client/customer, or combination of different people). To better explore the interpersonal dynamics at play, future research might examine the dyadic and team processes involved in who we help at work. It might be that if we direct helping strategies toward a colleague who is a “de-energizer” (i.e., difficult to interact with), then we do not see beneficial energy gains for the individual providing the help (Quinn, 2007; Schippers and Hogenes, 2011).

## Conclusion

This 1-week quantitative diary study, with ten repeated measurements, set out to investigate the relationship between use of work-related energy management strategies during the work day (i.e., prosocial, organizing, and meaning-related) and occupational well-being (i.e., job satisfaction and emotional exhaustion). In addition, this research paid special attention to chronic job demands as a boundary condition to the effectiveness of energy management.

Results of hierarchical linear modeling analyses showed that the use of prosocial energy management strategies, focusing on helping others at work, was positively linked to job satisfaction. Results further revealed that prosocial strategies can lessen exhaustion for those with high job demands. In contrast, organizing strategies are only beneficial, for both satisfaction and exhaustion, in jobs with low demands (and detrimental in jobs with high demands). Finally, reflecting on the meaning of one’s work might have a potentially backfiring effect for those without much job demand (i.e., pressure/challenge).

Overall, our findings suggest that employees’ use of certain strategies to manage their energy at work and the effectiveness of these strategies, is dependent on preexisting levels of job demand. Work-related strategies do not seem to provide clear respite from work (as compared to micro-breaks), and although some work-related strategies have the potential to activate one’s energy and improve a subjective sense of well-being (i.e., prosocial strategies), this depends on effectively *putting one’s resources to use in context*. Future research in larger and more heterogeneous samples is needed to arrive at a better understanding of micro-recovery processes during the working day and the boundary conditions of these processes.

## Author Contributions

HZ, TV, and CL designed the study and coordinated data collection. HZ analyzed the data. SP and HZ wrote the paper, and JdB provided critical feedback and presented initial findings at the Annual Meeting of the Society for Industrial and Organizational Psychology in 2016.

## Conflict of Interest Statement

The authors declare that the research was conducted in the absence of any commercial or financial relationships that could be construed as a potential conflict of interest.

## Footnotes

1 http://www.rug.nl/research/heymans-institute/organization/ecp/?lang=en
